# 12 Tips to Guarantee a Fragmented (Absolutely Terrible) Curriculum in a Time of Crisis

**DOI:** 10.15694/mep.2020.000208.1

**Published:** 2020-09-25

**Authors:** Constance Tucker, Abigail Kay

**Affiliations:** 1 Oregon Health & Science University; 2 Thomas Jefferson University Hospitals

**Keywords:** curriculum development, faculty development, failure, educational strategy, needs assessment, engagement

## Abstract

This article was migrated. The article was marked as recommended.

Medical education scholarship is filled with articles focused on rigorous curriculum design and innovation. In the midst of a public health crisis, the authors aim to provide a reflective guide to curriculum development focused on curriculum gone wrong. The authors propose twelve recommendations that will bring all educators closer to curricular failure.

## Introduction

“Success is stumbling from failure to failure with no loss of enthusiasm.” Winston Churchill

At this time of crisis, there is a tremendous amount of stress on academicians and therefore we wrote this, with a bit of humor, as a gift to our colleagues during this challenging time, with the hope that they may benefit from our failures.

In a time of world crisis, educators are often called upon to do the impossible - create a new course in a very short time and potentially deliver it in an entirely new format. This is difficult enough for an individual class, but can feel next to impossible with an entire course. It is our experience that these critical educational activities can lead to both innovative and scholarly work, but also can lead to a fragmented curriculum. We would like to focus our attention, not on the scholarly work, but rather on the fragmented curriculum. Sometimes you learn best not from rigorous scholarship but pure unadulterated failure. Using the approach of TRIZ to guide us, we will provide a surefire way to create a fragmented curriculum. You must begin by intentionally ignoring any scholarship on curriculum design and medical education, this is a key step in creating a fragmented curriculum. Although it may be tempting to read Curriculum Development for Medical Education: A Six-Step Approach, it is best avoided as it will most likely result in a non-fragmented curriculum ( Thomas *et al.*, 2015). For our gentle reader, who may not be familiar with this text, we will briefly summarize it but please do not commit this to memory or integrate any of these suggestions into practice if your aim is terrible curriculum.

Here is what we know: In Curriculum Development for Medical Education: A Six Step Approach, the authors explore a framework to develop, implement, and evaluate curriculum in the health professions, particularly medical education ( Thomas *et al.*, 2015). The six steps include (1) problem identification and general needs assessment, (2) targeted needs assessment, (3) development of goals and objectives, (4) educational strategies, (5) implementation, and (6) evaluation and feedback. The first step, problem identification and general needs assessment, focuses on identifying and defining a problem or need that can be addressed by a curricular activity. The second step is focused on targeted needs assessment in which the educators identify the differences between the ideal and actual characteristics of their targeted learners and their environment. This step asks who are the learners and what do these specific learners need.

**Figure 1.**
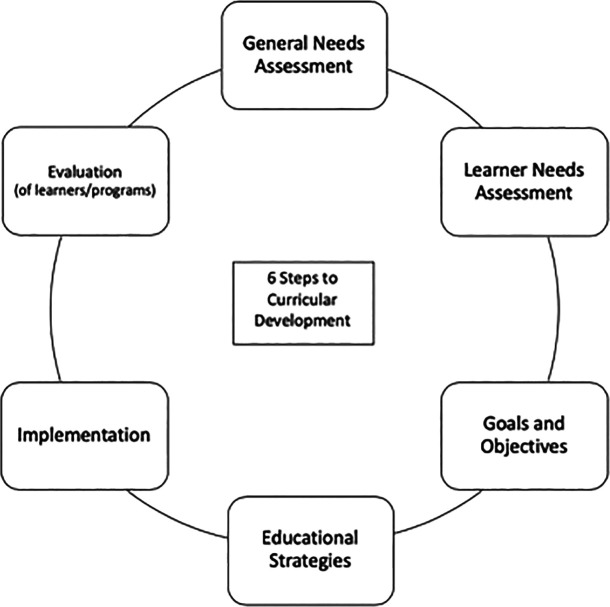
Six Step Approach to Curriculum Development

Figure 1. Adapted with permission from Thomas *et al.’s* (2015) Six Step Approach to Curriculum Development

Once the needs of learners are identified, the authors’ third step suggests that goals and SMART (i.e., specific, measurable, attainable, relevant, and time based) objectives are created to address the problem and the learners identified in step one and two. From well-written goals and objectives, curriculum developers can create content and methods, educational strategies (step four), that are aligned, engaging, and promote learning. In step five, implementation, the curriculum designers (i.e., you), obtain support for the curriculum. Support can occur through internal and external communications with diverse stakeholder groups, operations (i.e., scheduling, space, materials), and financing. The last step is to close the loop in curriculum development by using evaluation results to inform decision making about future curricular activities. Building on this work, the following twelve tips will help medical educators successfully create a fragmented curriculum.

## Twelve Tips

TIP 1

**Ignore the Problem** (General Needs Assessment):

Despite Thomas *et al.’s* (2015) reputable recommendations to do both general and targeted needs assessment, we encourage you to skip this step. Our experiences, should you trust us, have taught us that under no circumstances should you allow experts across disciplines to communicate, as this may result in active discussions about actual problems ( Altschuld and Kumar, 2010). If your desire is to keep your curriculum fragmented, do not talk about your curriculum with others. Thoughtful interactions and sharing has been known to cause innovative thinking ( Lipmanowicz and McCandless, 2014).

TIP 2

**Ask No (Zero) Questions About Needs** (General Needs Assessment):

Our previous tip highlighted the necessity of avoiding curricular conversations however, to underscore this point, we provide another useful pearl. While your mind may be curious about a curricular decision an administrator has made, avoid asking the educational administrator any challenging questions ( McKimm and Jones, 2018; Hammond, 2013). If you can, do not make eye contact, as they may read your non-verbal eye contact as demonstrating interest and think you have a question ( Cabaniss *et al.,* 2016). It is our experience that rather than question curricular intentionality, one should spend their spirited energy re-validating existing approaches to teaching.

TIP 3

**Work Only with Administrators when Developing Curriculum** (Targeted Needs Assessment):

Allow only administrators to determine curricular needs by excluding faculty and students from these discussions reinforcing the importance of hierarchy and sustaining hidden curriculum ( Lempp and Seale, 2004). Ideally, the administrators will have no background in education. When considering the learner, only envision the ideal learner. This is the learner who enjoys passively absorbing information, does not question how different topics, within and without a given lecture, are connected, and is happy to accept any content, even if it only applies to a narrow subset of a given patient population (e.g. only introduce the most expensive medications and do not discuss how uninsured patients can get access to life-sustaining medications) ( Graffam, 2007).

TIP 4

**If you work with faculty, Curricular Decisions Should Be Determined by Departmental Prowess** (Targeted Needs Assessment):

Allow the curricular needs to be decided by departmental prowess and territorial fighting; If there is a department with a faculty member whose research is obscure and only tangentially related to the topic, make certain that this is given additional time in the curriculum. Although this may necessitate multiple, large topics being condensed into one lecture, this can be overcome by giving the design of the condensed lecture to a junior faculty member who does not have any protected time to create educational material. Resort to a healthy respect of hierarchy, anger, fear, intimidation, and disillusion to reinforce the desired behavior ( Crowe, Clark, and Brugha, 2017).

TIP 5

**Avoid Curricular Maps at All Cost** (Goals & Objectives):

At all costs, avoid SMART goals. SMART goals have the disadvantage of focusing the learner and, potentially, of cultivating the student’s interest. This interest could result in a student doing further research on a topic, rather than limiting themselves to the provided content. Avoid alignment of the goals to the needs assessment or educational strategies as doing so could, yet again, result in a non-fragmented curriculum ( Al-Eyd *et al.,* 2018).

TIP 6

**Emphasize Non-Logical Relationships** (Goals & Objectives):

Organize the curriculum in non-sequential ways so that no logical relationship exists and so that students are less likely to make connections between the various lectures. This has the added benefit of making a specific subject seem so unattainable to a student that it will guarantee that they avoid specializing in that area of medicine.

TIP 7

**Pre-Packaged Curriculum is Ideal** (Educational Strategies):

Purchase a pre-packaged curriculum and disseminate to students; Pre-record all sessions and rely on modules. Ideally the speaker will not follow their slides and/or will provide excess details on their slides in order to make them more difficult to follow. Do not customize this curriculum to put it into context or in any way make it more meaningful to the students.

TIP 8

**Do NOT engage learners** (Educational Strategies):

As tempting as it is, avoid active learning at all costs; stress the importance of passive absorption of knowledge ( Peters *et al.,* 2019). Generations of successful clinicians have been created using this method and it would not make sense to mess with the success that this method has demonstrated ( Buja, 2019). Remind yourself, and others, how much you enjoyed this style of learning!

TIP 9

**Incentivize silos and provide no protected time** (Implementation):

Assure that technological, geographical, and physical barriers are in place to discourage experts from working together. Any online meeting (e.g. Zoom) should have no less than 50 participants, as this will prevent any thoughtful dialogue from occurring. This can be further enhanced by scheduling meetings during clinical hours, thus preventing clinicians from attending. Providing protected time can encourage faculty to be thoughtful about their talk, rather than allowing them to use the anxiety that comes with having to create a talk at the last minute, as fuel for their creativity and thoroughness on a given topic.

TIP 10

**Assume all faculty are fully competent to teach and assess** (Implementation):

Do not offer faculty development to support teaching as a competency ( Srinivasan *et al.*, 2011). If student feedback suggests the faculty member’s lecture is difficult to follow, it is key to not respond to the student’s negative mindset. This is especially true if the faculty member’s powerpoint slides are identical to the plastic overhead sheets that they used in 1987 ( Gadek, 1986).

TIP 11

**Only High Stakes Assessments should be used to determine learner competency** (Evaluation & feedback):

Use one single high stakes assessment activity to determine student learning, this will have the added benefit of simplifying the grading process ( Rudolph *et al.*, 2008). By providing no mid-way feedback through lesser stakes assessments, students will have the opportunity to strengthen their rote learning skills and will quickly forget the material, thus providing the mental space to “learn” new material.

TIP 12

**Discourage learner curiosity or feedback** (Evaluation & Feedback):

If you happen to receive feedback do not use the information to improve your curriculum, because learners do not know what they need. Similarly, discourage any conversations which might lead to curiosity ( Dyche and Epstein, 2011). Curiosity can lead to students pursuing the topic on their own and this can result in material being discussed, or questioned, which takes the focus away from your planned curricula of facts, techniques, and protocols.

## Conclusion

The twelve tips in this article are techniques, despite our best efforts, we learned through experience and recognize they contradict current scholarship of teaching and learning. We hope by sharing them, that others will benefit from our failures. While we acknowledge that the scholarship on curricular effectiveness is robust, educators seeking to amplify their effectiveness and efficiency while teaching in crisis should also allow themselves the gift of flexibility with their baseline level of perfectionism. We encourage you to embrace and reflect on the lack of achieving one’s normal level of success with humor and remember that perfection is the enemy of done.

## Take Home Messages

• In response to a pandemic, health professions educators were asked to deliver education in entirely new formats
• During such crises, humor can help educators to reflect on and benefit from failure
• The scholarship on effective curricular development is robust
• Important lessons can be learned from ineffective curriculum
• In curricular crisis, perfection is unnecessary

## Notes On Contributors

Constance R. Tucker, M.A., Ph.D. is the Vice Provost, Educational Improvement and Innovation at Oregon Health & Science University in Portland, Oregon. ORCID ID: https://orcid.org/0000-0002-6507-8832

Abigail Kay, M.A., M.D. is an Associate Dean of Academic Affairs and Undergraduate Medical Education at the Sidney Kimmel Medical College at Thomas Jefferson University Hospital in Philadelphia, Pennsylvania.

## Declarations

The author has declared that there are no conflicts of interest.

## Ethics Statement

The submitted practical tips manuscript does not require ethics approval as no human subjects research was conducted.

## External Funding

This article has not had any External Funding
