# Supplementary material for: 12 Tips to Guarantee a Fragmented (Absolutely Terrible) Curriculum in a Time of Crisis
Source: MedEdPublish (2016). 2020 Sep 25;9:208. [Version 1] doi: 10.15694/mep.2020.000208.1 (PMC10699399; doi:10.15694/mep.2020.000208.1)
Supplement: Peer review history

# Reviewer report: Reviewer response for version 1

**DOI:** 10.21956/mep.20126.r31059

**Published:** 2020-12-18

**Authors:** Ken Masters

**Affiliations:** 1 Sultan Qaboos University

This review has been migrated. The reviewer awarded 4 stars out of 5 A pithy and humorous review of the types of activities in curriculum design that can lead to disaster, while simultaneously pointing the reader in the right direction of the pertinent literature.While it is unlikely that any medical school has adopted all of these, I am sure that there are some there that everybody will recognise. One hopes the lessons will be learned.

Reviewer Expertise:

NA

No decision status is available

# Reviewer report: Reviewer response for version 1

**DOI:** 10.21956/mep.20126.r31058

**Published:** 2020-10-01

**Authors:** Adel Altawaty

**Affiliations:** 1 Libyan International Medical University

This review has been migrated. The reviewer awarded 4 stars out of 5 A devil's advocate approach. Good humorous writing that highlights common problems in curriculum design. Petty sure that authors and others have more in their minds to tell about issues leading to failure of curricular design. One of these is the curricular obesity which lead to cognitive overload of students.

Reviewer Expertise:

NA

No decision status is available

# Reviewer report: Reviewer response for version 1

**DOI:** 10.21956/mep.20126.r31057

**Published:** 2020-09-30

**Authors:** Rachel Shmuts

This review has been migrated. The reviewer awarded 5 stars out of 5 This is a delightful article that, while tongue-in-cheek and adversarial, actually does a lovely job of summarizing the key components of curriculum development while describing how to directly buck those guidelines. For those of us who have similarly slogged through curriculum reform at major medical education institutions, this is a light and relatable read that is both informative and validating.

Reviewer Expertise:

NA

No decision status is available

# Reviewer report: Reviewer response for version 1

**DOI:** 10.21956/mep.20126.r31056

**Published:** 2020-09-30

**Authors:** P Ravi Shankar

**Affiliations:** 1 American International Medical University

This review has been migrated. The reviewer awarded 4 stars out of 5 This is an interesting article and a ‘fresh’ perspective on curriculum design. The authors have examined common principles of curriculum design from the perspective of achieving a fragmented curriculum. The article will be of interest to all medical educators.

Reviewer Expertise:

NA

No decision status is available
